# Network Pharmacology and Data Mining Approach Reveal the Medication Rule of Traditional Chinese Medicine in the Treatment of Premenstrual Syndrome/Premenstrual Dysphoric Disorder

**DOI:** 10.3389/fphar.2022.811030

**Published:** 2022-06-21

**Authors:** Songlin Qu, Mingqi Qiao, Jieqiong Wang, Mingzhou Gao, Dan Chen, Shujing Li, Enhua Wei, Yinghui Guo

**Affiliations:** ^1^ College of Traditional Chinese Medicine, Shandong University of Traditional Chinese Medicine, Jinan, China; ^2^ Department of Science and Technology, Shandong University of Traditional Chinese Medicine, Jinan, China; ^3^ Department of the Graduate Student, Shandong University of Traditional Chinese Medicine, Jinan, China; ^4^ Laboratory of Traditional Chinese Medicine Classical Theory, Ministry of Education, Shandong University of Traditional Chinese Medicine, Jinan, China

**Keywords:** premenstrual syndrome, premenstrual dysphoric disorder, traditional Chinese medicine prescription, proprietary Chinese medicine, herbs, pharmacological mechanism, medication rule, network pharmacology

## Abstract

Premenstrual syndrome (PMS) is a common disorder that affects women of reproductive age. It is characterized by periodic mental and somatic symptoms such as irritability, depression, and breast pain during the luteal phase. Premenstrual dysphoric disorder (PMDD) is the most severe form of PMS. In recent years, the incidence of PMS/PMDD has been increasing year after year. However, due to the complex symptoms and ambiguous classification of PMS/PMDD, the limitations of present treatments, such as their poor efficacy rate, have become increasingly apparent. With its unique benefits such as syndrome differentiation and high cure rate, traditional Chinese medicine (TCM) has sparked new diagnosing and treating of PMS/PMDD. This study uses data mining methods, and statistical analysis revealed that Xiaoyao San and Chaihu Shugan San were the commonly used TCM to treat PMS/PMDD. A detailed investigation of regularly used single herbs revealed that most TCM is used as cold herbs that penetrate the liver meridian, with predominant bitter, sweet, and pungent flavors. The network pharmacology method analyzes the interactions between diseases, targets, and herbs. Meanwhile, the deep action targets and molecular mechanisms of 10 commonly used herbs for the treatment of PMS/PMDD are studied, revealing that it involves several ingredients, many targets, and different pathways. This interaction provides insight into the mechanism of action of TCM in the synergistic treatment of PMS/PMDD. It is now clear that we can begin treating PMS/PMDD with TCM using the target and mechanism revealed by the abovementioned findings in the future. This serves as an essential reference for future research and clinical application of TCM in the treatment of PMS/PMDD.

## Introduction

Premenstrual syndrome (PMS) is a common disorder with an incidence between 30 and 40% among women of childbearing age, in which mental, physical, and behavioral abnormalities occur regularly during the luteal phase and subside or disappear after menstruation, particularly mental symptoms such as mental tension, irritability or depression, breast pain, and other mental symptoms (Greene and Dalton, 1953). Premenstrual dysphoric disorder (PMDD) is a severe subtype of PMS that significantly affects the physical and mental health and working lives of women of childbearing age, lowers the quality of life and happiness index, and may even result in suicidal behavior, with an incidence of about 3%–8% (Ryu and Kim, 2015). In recent years, the incidence has gradually increased, and disease prevention and treatment are imminent. However, The Lancet recognized fluoxetine (Dimmock et al., 2000) as a first-line treatment in 2000. HalbreichU and other systematic reviews indicated that while these medications were more effective than placebo in treating PMDD, their response rate was less than 60%. Simultaneously, it was emphasized that the disease has subtypes and that precise placement would increase overall efficacy (Halbreich et al., 2006). Faced with shortcomings such as a poor cure rate and undefined subtypes of PMS/PMDD, TCM utilizes a multi-ingredient, multi-target, and multi-pathway approach that is superior in efficacy and has adverse effects on single-target or single-pathway drugs. TCM offers a novel, efficient, and targeted approach for diagnosis and treatment in this context.

In recent years, the use of TCM in the diagnosis and treatment of PMS/PMDD has gradually increased, the clinical treatment has demonstrated efficacy, and research into the mechanism of action has increased. She Xuhua treated patients with PMDD who had liver qi depression with Xiaoyao San, and this had a significant therapeutic effect on headache, breast pain, nodular pain, and limb swelling and significant differences in prolactin, estradiol, and progesterone levels before and after treatment (She, 2008). Wu Lilan used Chaihu-Shugan-San to successfully treat 40 patients with premenstrual syndrome with liver qi depression (Wu, 2010). Qiao Mingqi’s team validated the clinical cure rate of Jingqianping granule in the treatment of liver qi inversion syndrome in premenstrual syndrome through large-scale flow adjustment, and the clinical test demonstrated a favorable curative effect, with an effective rate of 96.04% (Qiao et al., 2002). Simultaneously, the Jingqianshu granule was developed to treat liver qi depression syndrome (Wang and Zhang, 2010). According to a review of the therapeutic prescriptions and unique Chinese patent medicines mentioned above, their essential components included single herbs such as *Bupleurum Chinensis DC* and *Radix Paeoniae Alba*. At the same time, experimental animal research into the antidepressant mechanism of single drugs is also increasing. On the other hand, the existing network of pharmacological studies on the depression mechanism of action of commonly used single drugs such as *Bupleurum Chinensis DC* and *Radix Paeoniae Alba* has revealed that they primarily contain active ingredients such as β-sitosterol, sitosterol, and flavonoids, with the primary targets being ADRA2A, ESR1, ADRA1B, and SLC6A4 (Chang et al., 2020). According to the research mentioned above, TCM pays more attention to the classification of treatment, precise use of drugs, and specific drugs used in the treatment of PMS/PMDD, and the therapeutic impact is impressive.

Although TCM is increasingly being used in diagnosing and treating PMS/PMDD, the efficacy, target, and molecular mechanism of each Chinese herbal medication or ingredient remain unknown. Systematic pharmacology is viewed as a promising approach for comprehending herbal formulations as it enables the systematic study of TCM at different scales, such as disease–gene–target–pathway active component interactions (Pei et al., 2016), to elucidate the material basis and mechanism of action of TCM or components (Zhang R. et al., 2019; Zhang W. et al., 2019). By using the systematic pharmacology method, this study comprehensively analyzed the existing literature on the diagnosis and treatment of PMS/PMDD in TCM; classified the commonly used Chinese medicine prescriptions, proprietary Chinese medicine, and single medicine; and systematically predicted and analyzed the complex relationship between target, TCM, and disease, providing ideas and a theoretical basis for follow-up, TCM selection, new drug development, and mechanism research.

## Materials and Methods

### Datasets Collection and Analysis

**FIGURE 1:**
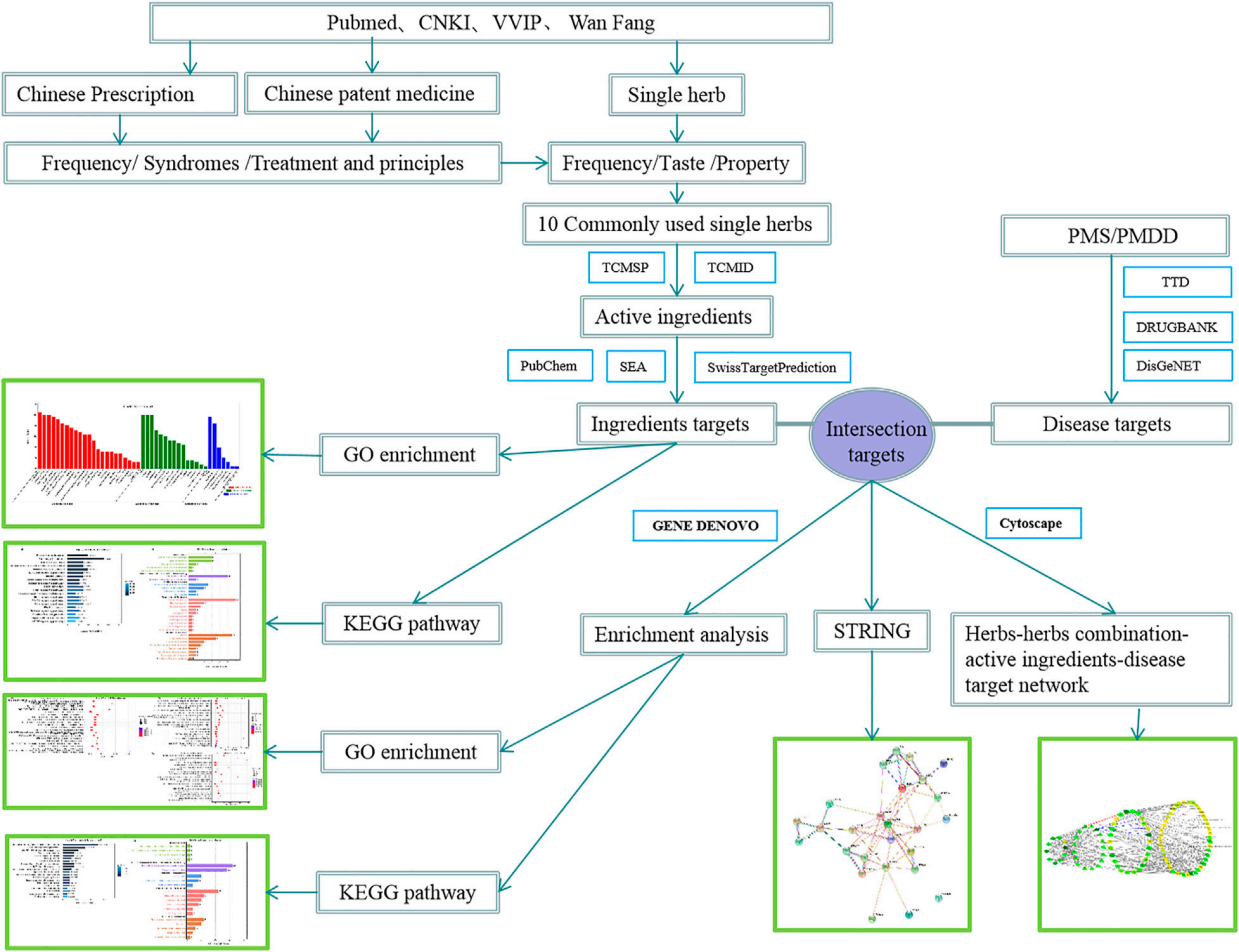
Workflow figure of this study.

**FIGURE 2:**
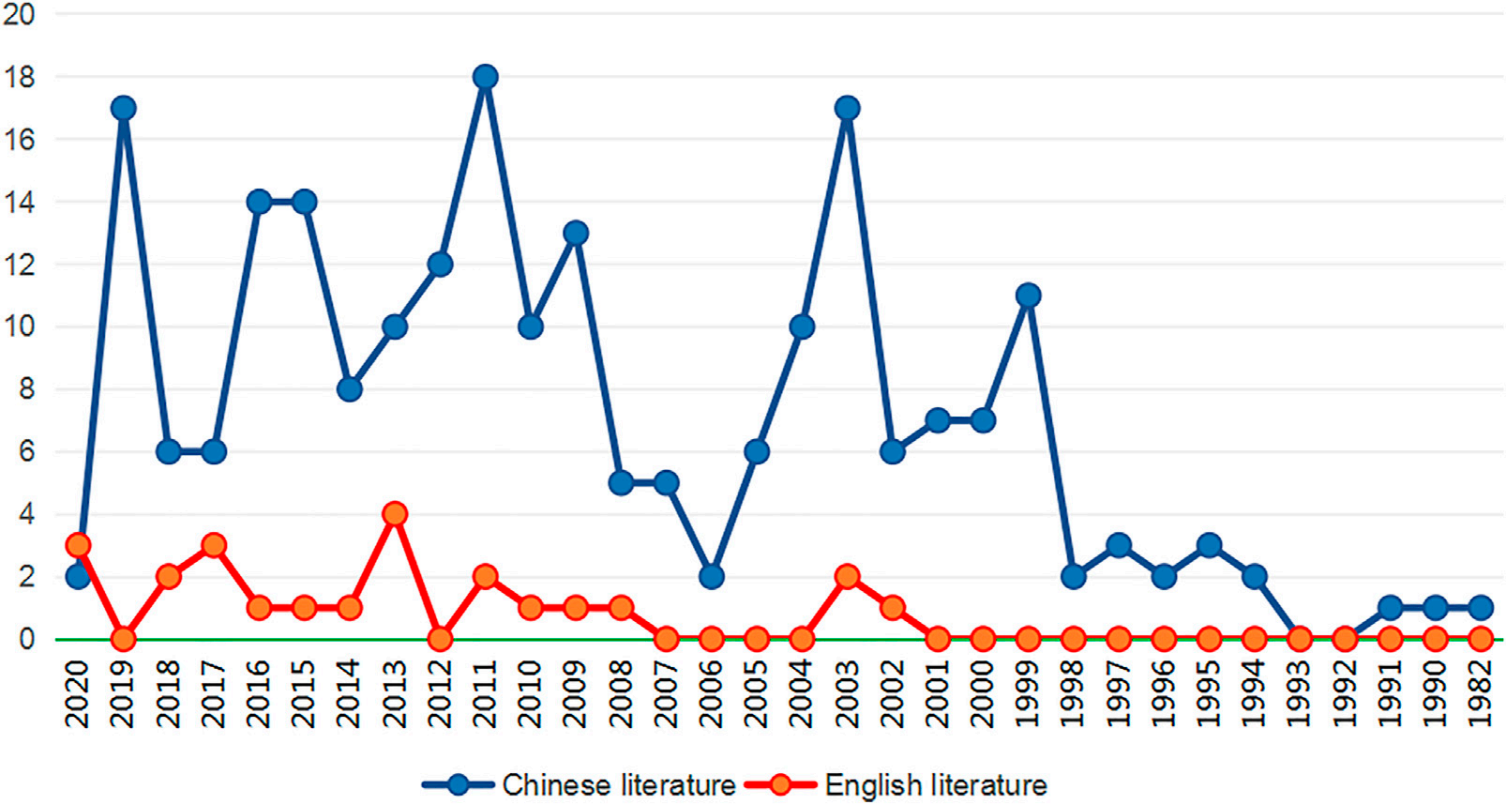
Year distribution table of TCM-related literature on PMS/PMDD. The abscissa indicates the year of publication (years of publication), and the ordinate indicates the number of publications in a specific year (number of publications). The blue line represents the Chinese literature on PMS/PMDD Chinese medicine treatment, and the red line represents the English literature on PMS/PMDD Chinese medicine treatment.

The overall flow figure of this study is shown in Figure 1. Literature sources: we used the terms “Premenstrual dysphoric disorder,” “Premenstrual syndrome,” “PMDD,” “PMS,” “traditional Chinese Medicine,” and “treatment” to search for relevant information in the China knowledge Network (CNKI), Wan Fang, VVIP, and PubMed databases. From the beginning of the year to the present, Note Express document management software screened all the related literature in the database, resulting in the acquisition of relevant articles. Figure 2 shows the specific year distribution.

Criteria for data inclusion and exclusion: inclusion criteria: (1) Literature contains complete and precise clinical prescriptions for various syndrome types of premenstrual syndrome/premenstrual dysphoric disorder and reports on the pharmacological mechanism of herbs commonly used in the treatment. (2) For the summary literature, (1) is taken as the standard. The original data are explored based on the references, and qualified data are collected. Exclusion criteria (1) relevant individual clinical cases; (2)those having unclear herb composition and insufficient clinical treatment data; and (3) clinical western medicine treatment and those inconsistent with the keywords.

Prescription screening and single herb name standardization: A total of 63 prescriptions were selected based on the inclusion and exclusion criteria. The names of the herbs appearing in prescriptions are standardized in the People’s Republic of China Pharmacopoeia (2020 edition) and the textbook “Traditional Chinese Medicine” of the 13th five-year Plan of higher Education in the Traditional Chinese Medicine Industry. “*Raw Radix Paeoniae Alba*” is uniformly standardized as “*Radix Paeoniae Alba*” and “*Concocted Rhizoma Cyperi* " standardized as “*Rhizoma Cyperi*.” The Chinese name, used part, English name, and Latin name of herbs are shown in Supplementary Table S1.

Data entry and analysis: the selected and standardized prescriptions are inputted into the Excel form. The two authors were responsible for validating the input data one by one to assure data mining accuracy. Statistics were built on the frequency of use, syndromes, and treatment associated with commonly used TCM prescriptions and Chinese patent medicines, ranked based on the frequency of occurrence of TCM in all prescriptions, and sorted out based on the nature, taste, and meridian tropism of commonly used herbs.

### Screening of Active Ingredients in 10 Herb Sorting, Identification of PMS/PMDD Disease Targets, and Herb Targets

To collect the chemical constituents of a single medicine, we logged on to the TCMSP platform (http://tcmspw.com/tcmsp.php) (Ru et al., 2014). The TCMID database (http://www.megabionet.org/tcmid/) was used to supplement the search for information on TCM (Guo et al., 2019), lacking in the TCMSP database, and the chemical ingredients of commonly used single herbs were obtained. ADME (Lucas et al., 2019) was used to screen the data, and the active chemical ingredients were identified based on their bioavailability (OB) ≥ 30% and drug similarity (DL) ≥ 0.18. Multiple items were deleted from this list to obtain ten different types of active ingredients of herbs. The ingredient action target (Gfeller et al., 2014) was identified using the SwissTargetPrediction database (http://www.swisstargetprediction.ch/) and the SEA data database (https://sea.bkslab.org/). The duplicate targets were excluded. The Uniprot database (https://www.uniprot.org/) was used to standardize the target proteins in preparation for the subsequent construction of the herb–herb combination–active ingredient–disease target network. We used the TTD database (http://bidd.nus.edu.sg/group/cjttd/), the DrugBank database (https://go.drugbank.com/), and the DisGeNET database (https://www.disgenet.org/) to screen the disease targets by searching the keyword “Premenstrual Dysphoric Disorder” and “Premenstrual Syndrome” (Chen et al., 2002; Piñero et al., 2017; Wishart et al., 2018), and the Uniprot database (https://www.uniprot.org/) was also used to standardize the target proteins.

### Construction of Herb–Herb Combination–Active Ingredient–Disease Target Network

The standardized active ingredient targets were compared with disease targets to obtain intersection targets, and then the intersection targets were compared with the respective targets of ten single herbs. According to the intersection targets contained in every single herb, the herbs containing common intersection targets were grouped into a group (the number of herbs contained in every combination ≥3 was included in the study), and a total of 17 combinations of herbs were obtained, See Supplementary Table S8 for details. The Network of the herb–herb combination–active ingredients–disease target was constructed using Cytoscape3.7.2 software. The data were imported into Cytoscape to obtain a visual network of the herb–herb combination–active ingredient–disease target, and the “Tools-Network Analysis-Analyze Network” was selected to calculate the degree of nodes in the Network. Meanwhile, select “Style - Node” to render the degree and node color of the target network. The degree value defines the core target, ingredient, herbs, and herb combination (Shannon et al., 2003).

The String database (https://string-db.org/) was used to predict the interaction between target proteins. The target of joint prediction between herbs and disease was imported into the String database, and the study species “*Homo sapiens*” was selected to obtain the target protein interaction relationship (Doncheva et al., 2019).

### Herb–Herb Combination–Active Ingredient–Disease Target Analysis Using GO Enrichment and KEGG Pathway Analysis

Using the GENE DENOVO (https://www.omicshare.com/tools/) online platform for high-throughput GENE function analysis to clarify the biological characteristics of the herb–herb combination, the active ingredients, and the disease intersection target and obtain the analysis results of biological processes (BP), cellular components (CC), and molecular function (MF) belonging to GO enrichment, we performed Kyoto Encyclopedia of Genes and Genomes (KEGG) pathway analysis for intersection targets. Then, we selected the option of “GO enrichment analysis” and “KEGG pathway analysis” separately in the platform and imported the intersection targets into the platform according to the requirements of sample files. Select “*Homo sapiens* (GRCH38.p13)" for version/species/type and keep the default options for other items. After submitting the analysis, the corresponding GO enrichment and KEGG pathway analysis results were obtained. (Liang et al., 2019; Wu et al., 2019).

### Screening of the Intersection Targets of Ten Commonly Used Herbs, GO Enrichment, and KEGG Pathway Analysis

Due to a large number of different types of herbs that could not be selected simultaneously, herbs were classified into three categories (bitter, sweet, and bitter-sweet) using the Venn diagram software (http://bioinformatics.psb.ugent.be/webtools/Venn/) based on their taste: group 1 (bitter taste: *Bupleurum Chinensis DC*, *Paeoniae Radix Alba*, *Rhizoma Chuanxiong*, *Fructus Aurantii*, and *Radix Curcumae*), group 2 (sweet taste: *Angelicae Sinensis*, *Poria cocos*, and *Astragalus membranaceus*), and group 3 (bitter-sweet: *Rhizoma Cyperi*, *Rhizoma Atractylodis and Macrocephalae*). The three sets of intersection targets were combined to obtain the intersection. The intersection target formed by this junction is the target shared by the ten Chinese medicines. The target data were imported into the GENE DENOVO platform for the KEGG pathway and GO enrichment analyses. The method of analysis is identical to that described previously.

## Results

### Analysis of Frequency of Use, Corresponding Syndromes, and Treatment Principles of Commonly Used Chinese Medicine Prescriptions in the Clinical Treatment of PMS/PMDD

TCM diagnosis and treatment of PMS/PMDD are based on syndrome differentiation, with different prescriptions utilized to address the symptoms of each syndrome type. From the beginning of the year to the present, 1,003 related literature reports were retrieved from the China National Knowledge Infrastructure (CNKI), Wanfang database, China Science and Technology Journal Database (VIP), and PubMed database. Seventy-six and 30 literature reports were retrieved from the Wanfang and Wei Pu databases. 886 articles and 433 articles were obtained from the PubMed database. The Note Express document management software retrieved a total of 244 valid documents. Figure 2 describes the year distribution table of TCM-related literature on PMS/PMDD.

There were 21 common prescriptions and 52 self-made prescriptions identified for the treatment of PMS/PMDD, and the common prescriptions used in the clinical treatment of PMS/PMDD with a frequency ≧5, and their related treatment syndromes and treatment methods were compiled. Overall, the study discovered that Xiaoyao San, Chaihu-Shugan-San, Xuefu Zhuyu Decoction, and Danzhi Xiaoyao San are used more frequently to treat PMS/PMDD, with the frequency of use being 48, 34, 24, and 19 times, respectively. The treatment syndromes included liver qi stagnation, qi stagnation, blood stasis, liver stagnation to transform fire, etc. As shown in Table 1, the therapeutic approaches aimed to soothe the liver and relieve depression, clearing heat and dissipating fire, regulating qi, and promoting blood circulation. Among them, Xiaoyao San and Chaihu Shugan San both have the combination of *Bupleurum Chinensis DC* and *Paeoniae Radix Alba* and dispersing and collecting. The treatment approach is primarily focused on relieving liver depression and nourishing liver blood. The use of *Gardenia jasminoides Ellis, Scutellaria baicalensis Georgi*, and other heat-clearing and detoxifying herbs enhances the power of heat-clearing and eliminating fire. The clinically representative prescriptions include Danzhi Xiaoyao San and Longdan Xiegan Decoction to treat liver stagnation and clear the fire. The qi stagnation and blood stasis syndrome treatment use *Semen persicae* and *Carthamus tinctorius*. The primary blood-activating medications are generally compatible with *Platycodon grandiflorus*, *Fructus aurantii*, and other qi-invigorating medications. Blood circulation to alleviate blood stasis is combined with the circulation of qi to dissipate qi stagnation and blood stasis. The typical prescription is Xuefu Zhuyu Decoction. *Rehmanniae Radix*, *Cornus Officinalis Sieb*, *Rhizoma Dioscoreae*, *Lycii Fructus*, and other yin nourishing medicines are frequently utilized for Yin deficiency syndrome. *Pinellia ternata*, *Rhizoma atractylodis Macrocephalae*, and *GasTCMLIBodiaelata Bl* for phlegm-fire disturbance syndrome are used as expectorants.

**TABLE 1:** Frequency of commonly used prescriptions for clinical treatment of PMS/PMDD, corresponding syndromes, and treatment rules (frequency ≥5).

Prescription	Frequency of use	Corresponding syndromes	Treatment rule
XiaoyaoSan	48	Stagnation of liver qi	Dispersing stagnated liver qi for relieving qi stagnation, nourishing the blood, and strengthening the spleen
Chaihu-shugan-San	34	Stagnation of liver-qi	Dispersing stagnated liver qi for relieving qi stagnation, promoting qi circulation, and relieving pain
Xiaochaihu decoction	11	Stagnation of liver qi	Harmonizing Shaoyang
Xuefu Zhuyu decoction	24	Qi stagnation and blood stasis	Promoting the blood circulation and removing blood stasis, regulating qi, and relieving pain
Danzhi Xiaoyao San	19	Liver depression forming fire	Dispersing stagnated liver qi for relieving qi stagnation, strengthening the spleen, clearing heat, and suffocating
Longdan Xiegan decoction	13	Liver depression forming fire	Clearing excess liver and gallbladder fire, clearing dampness, and heat of liver meridian
Yiguan Jian decoction	16	Liver-Kidney Yin deficiency	Nourishing yin and dispersing stagnated liver qi
Guipi decoction	9	Heart-spleen deficiency	Nourishing qi and replenishing blood, invigorating spleen, and nourishing heart
Jiangu decoction	9	Sinking of qi due to spleen deficiency	Nourishing spleen and consolidating blood, oozing dampness, and stopping diarrhea
Buzhong Yiqi decoction	6	Sinking of qi due to spleen deficiency	Tonifying middle-Jiao and qi, raising Yang, and lifting prolapsed zang-fu organs
Qiju Dihuang decoction	7	Hyperactivity fire due to yin deficiency	Nourishing the kidney and liver and improving eyesight
Zhibo Dihuang decoction	5	Hyperactivity fire due to yin deficiency	Nourishing Yin for lowering fire
HuanglianWendan decoction	6	Phlegm-fire attacking upward	Clearing heat and dampness, regulating the flow of qi for eliminating phlegm, and harmonizing stomach and gallbladder
Wendan decoction	5	Phlegm-fire attacking upward	Regulating the flow of qi for eliminating phlegm, harmonizing stomach, and gallbladder

Note: Please refer to Supplementary Table S2 for details of all herbs (taste, property, and meridians) contained in the prescriptions listed in the table.

### Proprietary Chinese Medicines Commonly Used in the Clinical Treatment of PMS/PMD and Their Corresponding Syndromes and Treatment Principles

This study highlights the most often used Chinese patent medications for the clinical treatment of PMS/PMDD (use frequency ≧2), the corresponding syndromes, and treatment techniques, as shown in Table 2. Because there are not many Chinese patent medicines for the treatment of PMS/PMDD, their specific frequency is not analyzed. The findings revealed that the currently available Chinese patent medications for the treatment of PMS/PMDD primarily target liver qi stagnation, liver qi up heavy, qi stagnation, blood stasis, and spleen deficiency. The majority suffers from liver qi stagnation, and Jingqianshu granules are commonly used clinically. Shuyu Capsules, Yue’an Decoction, and Jingqian’an tablets are Chinese patent medications used to treat liver-qi stagnation syndrome. Jingqianping granules and Baixiangdan capsules are both Chinese patent drugs frequently used to treat PMS/PMDD liver qi inversion syndrome. They are transformed from Chaihu-Shugan-San. They are valuable because they may calm the liver, regulate qi, alleviate swelling and pain, and simultaneously treat PMS/PMDD. Wang Qingren, a physician during the Qing Dynasty, invented Xuefu Zhuyu decoction. It promotes blood circulation, dispels blood stasis, soothes the liver, and regulates qi, while causing no harm to qi or blood. It is more readily taken and absorbed. In addition, there are herbs such as *Paeoniae radix Alba*, *Rhizoma atractylodis Macrocephalae*, and *Rhizoma cyperi* for YueqianShu Jian, which can relieve the liver and depression, while regulating qi and invigorating the spleen, successfully treating PMS disorders.

**TABLE 2:** Commonly used Chinese patent medicines in the clinical treatment of PMS/PMDD, corresponding syndromes, and treatment rules.

Chinese patent medicines	Corresponding syndrome	Treatment rule
Jingqianshu granule	Liver qi stagnation	Dispersing stagnated liver qi for relieving qi stagnation, regulating qi, and dispersing knot
Shuyu capsules		
Yue’an Jian		
Jingqian’an tablets		
Kunyue’an tablets		
Shugan jieyu capsules		
Jingqianping granule	Liver-qi invasion	Suppressing hyperactive liver, regulating qi, eliminating distension, and relieving pain
Baixiangdan capsules		
Xuefu zhuyu capsules	Qi stagnation and blood stasis	Regulating qi, promoting blood circulation, and removing blood stasis
Yueqianshu Jian	Depressed liver and deficient spleen	Dispersing stagnated liver qi, regulating qi, strengthening spleen, and tranquilizing mind

### The Active Ingredients, Pharmacological Effects, and Mechanism of Herb Action Frequently Used to Treat PMS/PMDD

**FIGURE 3:**
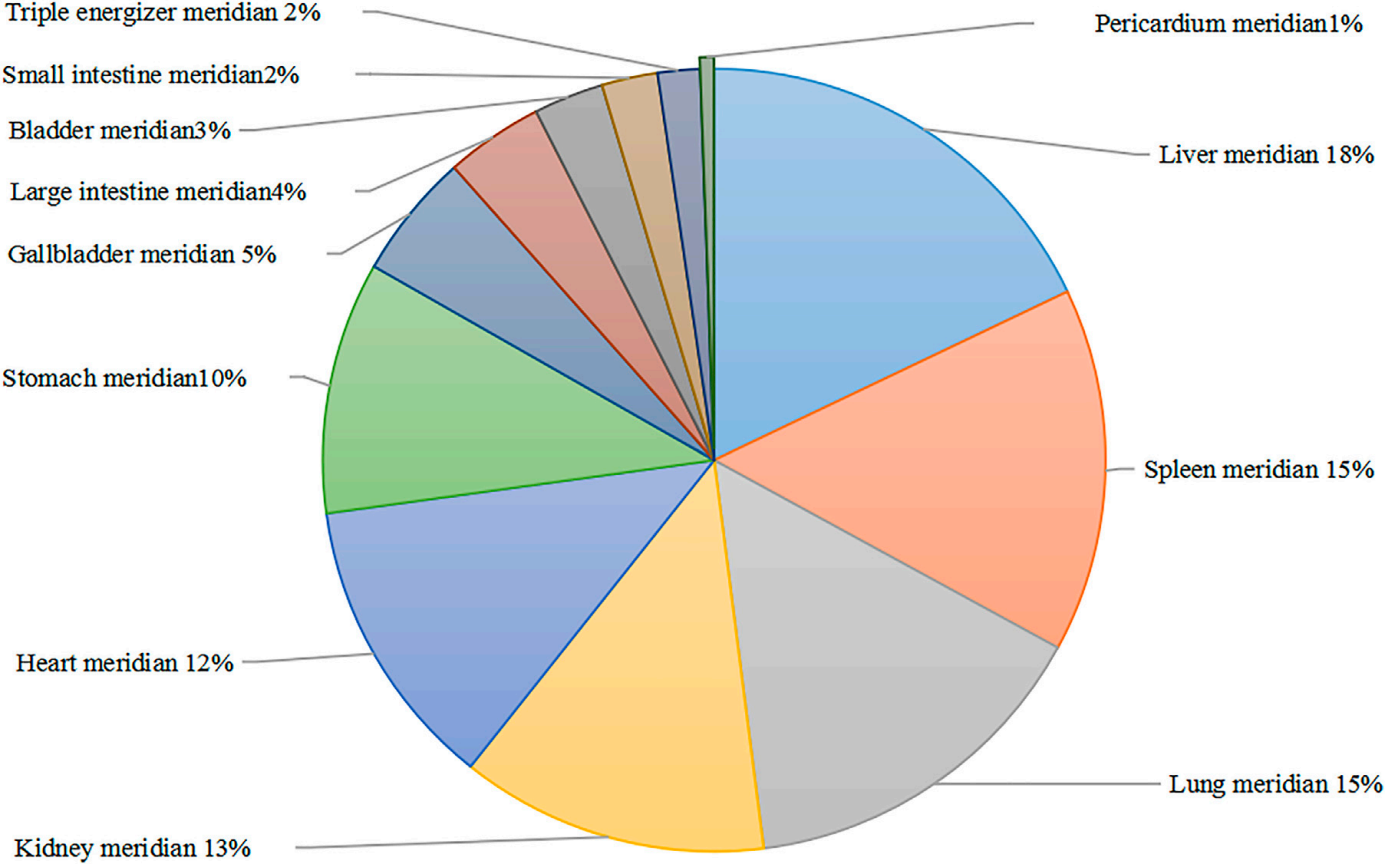
Frequency ratio of herb attribution meridian. Different colored plates represent different meridians.

Statistical analysis was performed on the properties, taste, and meridian status of herbs comprising the prescriptions in Table 1 and Supplementary Table S2. The data revealed that bitter medications are the most often utilized type of medicine (32.38%), followed by sweet and pungent medicines (30.48% and 21.90%). The sum of the three medicinal tastes was 84.76%. Cold Chinese medicine was the most common (42.19%), followed by warm Chinese medicine (35.94%), as shown in Table 3. In terms of the frequency of herbs, liver meridian (31 times), spleen meridian (26 times), lung meridian (26 times), kidney meridian (22 times), heart meridian (21 times), stomach meridian (18 times), gallbladder meridian (9 times), large intestine meridian (7 times), bladder meridian (5 times), small intestine meridian (4 times), triple energizer meridian (3 times), and pericardial meridian (1 time) are the most frequently mentioned. According to the information provided above, the herbs for the treatment of PMS/PMDD account for the most significant proportion of the liver meridian, which is 18%; the two meridians of the spleen and lung account for 15%; the three meridians of the liver, spleen, and lung account for 48%. The proportion of the rest of the specific return to the meridian is shown in Figure 3.

**TABLE 3:** Frequency statistics of the taste and property of herbs used in the treatment of PMS/PMDD.

Drug taste	Frequency of use	Proportion (%)	Drug property	Frequency of use	Proportion (%)
Bitter	34	32.38	Cold	27	42.19
Sweet	32	30.48	Warm	23	35.94
Spicy	23	21.90	Flat	12	18.75
Acid	7	6.67	Plain	2	3.13
Salty	3	2.86			
Light	3	2.86			
Astringent	3	2.86			

The frequency of use, properties, main ingredients, and pharmacological effects of several single medicines regularly used in the treatment of PMS/PMDD is summarized, and a single-medicine with a frequency of use ≧40 was selected for further investigation. Table 4 presents the specific content. According to the findings of the study, there are primarily ten common single herbs used in the treatment of PMS/PMDD (use frequency ≧40), with *Bupleurum Chinensis DC*, *Radix Paeoniae Rubra*, *Rhizoma Cyperi*, *Rhizoma Atractylodis Macrocephalae*, and so on rotundus corresponding saikosaponins, total glucosides of paeony, flavonoids, and volatile oils having the main pharmacological effects. The most critical pharmacological effects include antidepressants, immune enhancement, antiaging, anti-inflammatory and analgesic, and prevention of thrombosis. These benefits are achieved through various pharmacological pathways.

**TABLE 4:** Frequency, property, flavor, main ingredients, pharmacological effects, and meridians of common herbs used in the treatment of PMS/PMDD (Frequency≥ 40).

Herbs	Frequency of use	Property and flavor	Main ingredients	Pharmacological effects	Meridians
*Bupleurum chinensis DC*	196	Bitter, spicy, and cold	Bukosaponins, volatile oil, polysaccharides, flavonoids, and sterols	Inhibit depression, prevent convulsion, prevent epilepsy, prevent tumor, prevent thrombosis, etc.	Liver, gallbladder, triple energizer, and pericardium
*Radix Paeoniae Alba*	172	Sweet, spicy, and warm	Total glycosides of paeoniflorin and paeoniflorin	Inhibition of depression, blood pressure, anti-inflammatory, improve immunity, neuroprotection, etc.	Liver and spleen
*Angelica Sinensis*	169	Sweet, spicy, and warm	Ligustilide, ferulic acid	Antitumor, antioxidation, delay aging, reduce radiation, improve immunity, analgesia, etc.	Liver, heart, and spleen
*Poria cocos*	144	Sweet, light, and flat	Poria cocos	Inhibit tumor, improve immunity, hypoglycemic, sterilization, anti-inflammatory, etc.	Heart, spleen, and kidney
*Rhizoma Atractylodis Macrocephalae*	124	Bitter, sweet, and warm	Volatile oils, polysaccharides, and amino acids	Anti-tumor, diuresis, sterilization, delaying aging, regulating blood sugar, improving immunity etc.	Spleen and stomach
*Rhizoma Chuanxiong*	90	Bitter, spicy, and warm	Ligustilide, ferulic acid, and ligustrazine	Prevent thrombosis, calcium antagonism, tumor prevention, analgesic and spasmolytic prevent platelet aggregation, etc.	Liver, gallbladder, and pericardium
*Fructus Aurantii*	61	Bitter, spicy, sour, and cold	Volatile oils, alkaloids, coumarins, and flavonoids	Regulating gastrointestinal motility, antitumor, reducing blood lipid, anti-inflammation analgesia, etc.	Spleen and stomach
*Rhizoma Cyperi*	58	Slightly bitter, sweet, spicy, and flat	Volatile oils, flavonoids, triterpenes, and steroidal saponins	Reduce blood pressure, regulate menstruation and relieve pain, anti-inflammation and sterilization, prevent thrombosis, etc.	Liver and triple energizer
*Radix Curcumae *	40	Bitter, spicy, and cold	Volatile oil, polysaccharide, a small number of trace elements, and β-sitosterol	Cholagogic, anti-platelet aggregation, central nerve inhibition, protection of gastric mucosa, cancer prevention, etc.	Liver, heart, and lung
*Astragalus membranaceus*	40	Sweet and warm	Saponins, sucrose, polysaccharides, and various amino acids	Delay senescence, suppress stress, enhance immunity, regulate blood sugar, reduce blood pressure etc.	Lung and spleen

### Analysis of the Active Ingredients of Herbs Commonly Used in the Treatment of PMS/PMDD Based on Network Pharmacology, Collected Targets of Active Ingredients, and Disease

The active ingredients of ten types of single medications typically used in the treatment of PMS/PMDD were obtained using the TCMSP and TCMID databases, yielding 9,782 active ingredients. After screening and deleting duplicates, 105 active ingredients of herbs, including (+)-anomalin, beta-sitosterol, and ferulic acid (FA), were found using bioavailability OB ≥ 30% and drug similarity DL ≥ 0.18), as shown in Supplementary Table S3. A total of 105 active ingredients of herbs were imported into the SwissTargetPrediction database and SEA database. After removing the duplicates, 1,285 targets, including muscarinic acetylcholine receptor M2 (CHRM2), ESR1, and beta-1 adrenergic receptor (ADRB1), were obtained (Supplementary Table S4). The TTD, Drugbank, and DisGeNET databases were used to predict PMS/PMDD disease targets. After eliminating the duplicates, a total of 82 active ingredient targets, including ESR1, ESR2, and cytochrome P450 3A4 (CYP3A4), were obtained (Supplementary Table S5).

### Construction of the Interaction Network of Herb Active Ingredients and Disease Intersection Targets and Proteins

**FIGURE 4:**
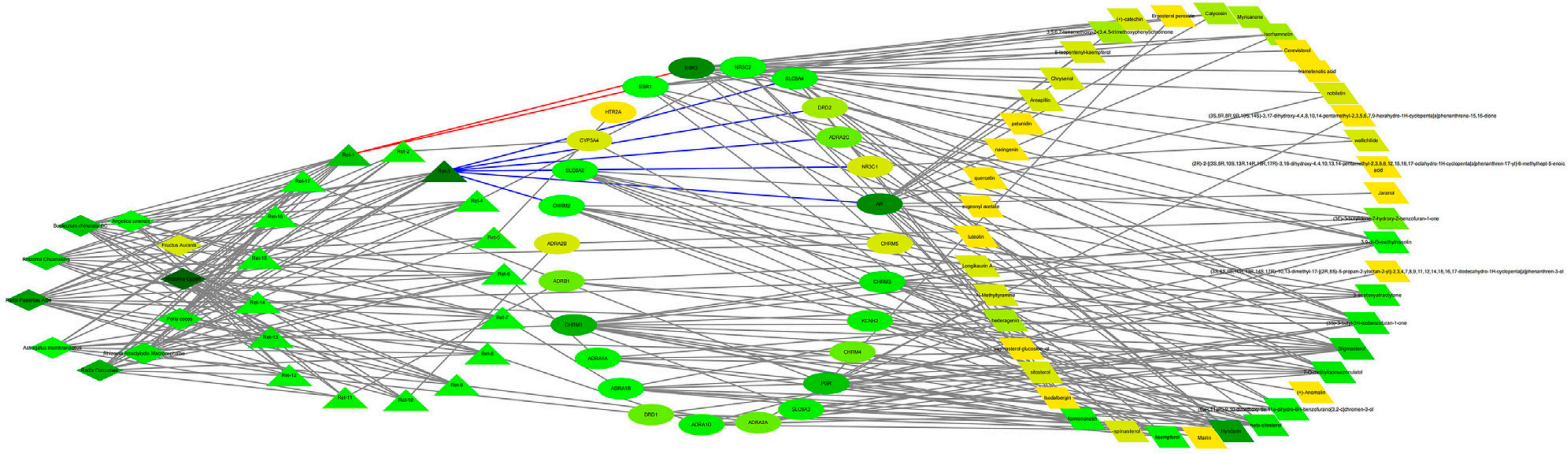
Herb–herb combination–active ingredient–disease target network diagram. See Supplementary Tables S6, S7 for details. Ret stands for different combinations of herbs, as shown in Supplementary Table S8. Ret-1 contains ten herbs corresponding to two targets, ESR1/ESR2 (red line). Ret-3 contains the most significant number of common targets (blue line). The color gradually darkens (yellow–light green–dark green), and the degree of freedom (degree) increases.

Through the target intersection of active ingredients and diseases of PMS/PMDD, we obtained CHRM1/CHRM2/CHRM3/CHRM4/CHRM5, SLC6A2/SLC6A3/SLC6A4, ADRA1A/ADRA1B/ADRA1D/ADRA2A/ADRA2B/ADRA2C, ESR1/ESR2, and DRD1/DRD2 and other 26 intersection targets, mainly belonging to solute carrier family 6, cholinergic receptor, adrenergic receptor, estrogen receptor, and dopamine receptor families (Supplementary Table S6). The abovementioned data suggest that herbs may play a role in the treatment of PMS/PMDD by intervening in these core targets (as shown in Figure 4, the color gradually darkens (yellow–light green–dark green), and the degree of freedom increases, the more likely it is to become a core protein). We also identified the single medicines corresponding to the 26 intersection targets and their corresponding specific ingredients and the 42 candidate core ingredient information. The herb’s most commonly used active ingredients were stigmasterol, kaempferol, hyndarin, etc. (Supplementary Table S7). Combining Chinese medicines with common intersection targets (the number of common intersection targets in each combination is ≥ 3) resulted in 17 combinations. For specific combinations, see Supplementary Table S8. The information obtained from the combination of herbs, active ingredients, and disease targets was imported into the Cytoscape software to construct a network diagram of herb–herb combination–active ingredients–disease targets, as shown in Figure 4. On observing all of the active ingredients in herbs, the ESR2, AR, CHRM1, and PGR intersection targets appeared to be the most frequently seen. RET-1 combines all ten single herbs, acting on two overlapping targets, namely, ESR1/ESR2 (see the red line in Figure 4). When used in conjunction with other RET-3 groups, the RET-3 group (except *Fructus Aurantii*) includes AR, CHRM2, and other seven intersection targets, with the most significant number of intervention intersection targets (Figure 4 blue line mark). There are several intersecting targets for PMS/PMDD intervention in stigmasterol, kaempferol, hyndarin, and other active ingredients. These ingredients may play an essential regulatory role in treating PMS/PMDD.

**FIGURE 5:**
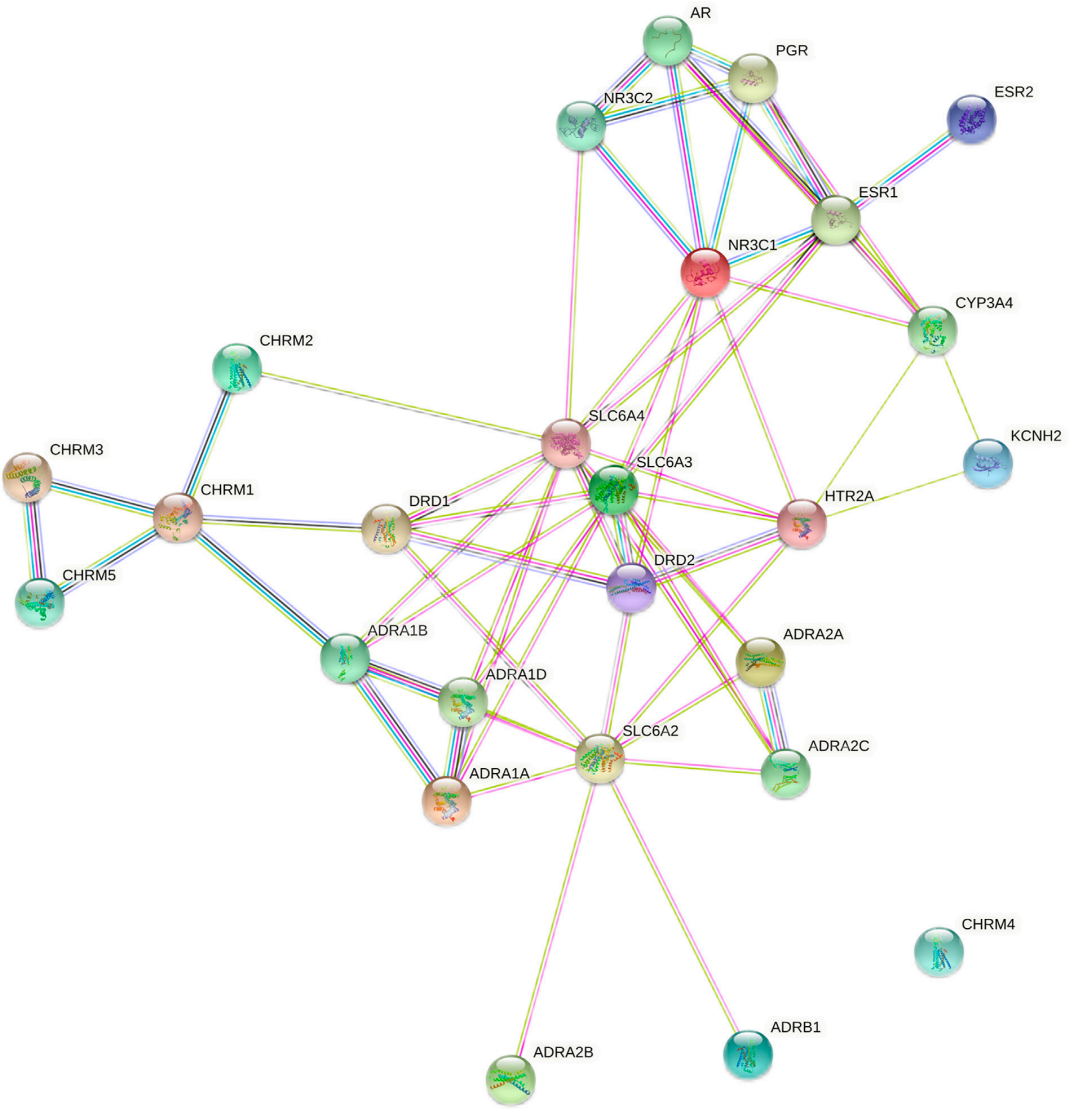
Protein interaction network diagram for TCM active ingredient–disease intersection targets. The greater the number of adjacent nodes, the greater the probability of becoming a core gene.

The STRING database was used to predict the interaction relationship between the intersecting target proteins by importing the 26 intersecting targets to obtain a network diagram of the interaction between the targets, as shown in Figure 5. There were 64 interaction relationships in the target protein interaction network. The average node degree value was 4.85, and the average local clustering coefficient was 0.553. The greater the number of adjacent nodes, the more likely it becomes a core protein. The core proteins mainly included SLC6A4, SLC6A3, SLC6A2, NR3C1, ESR1, and HTR2A. These core proteins (as shown in Figure 5) play an essential role in the regulation of the PPI network, but they are not the existing core intervention targets of the traditional Chinese medicine (as shown in Figure 4), suggesting that we can try to start from the abovementioned targets and use these targets as potential therapeutic targets of PMS/PMDD for subsequent research and development of targeted drugs against these targets.

### GO Enrichment Analysis of the Active Ingredients of Herbs-Disease Intersection Target

**FIGURE 6:**
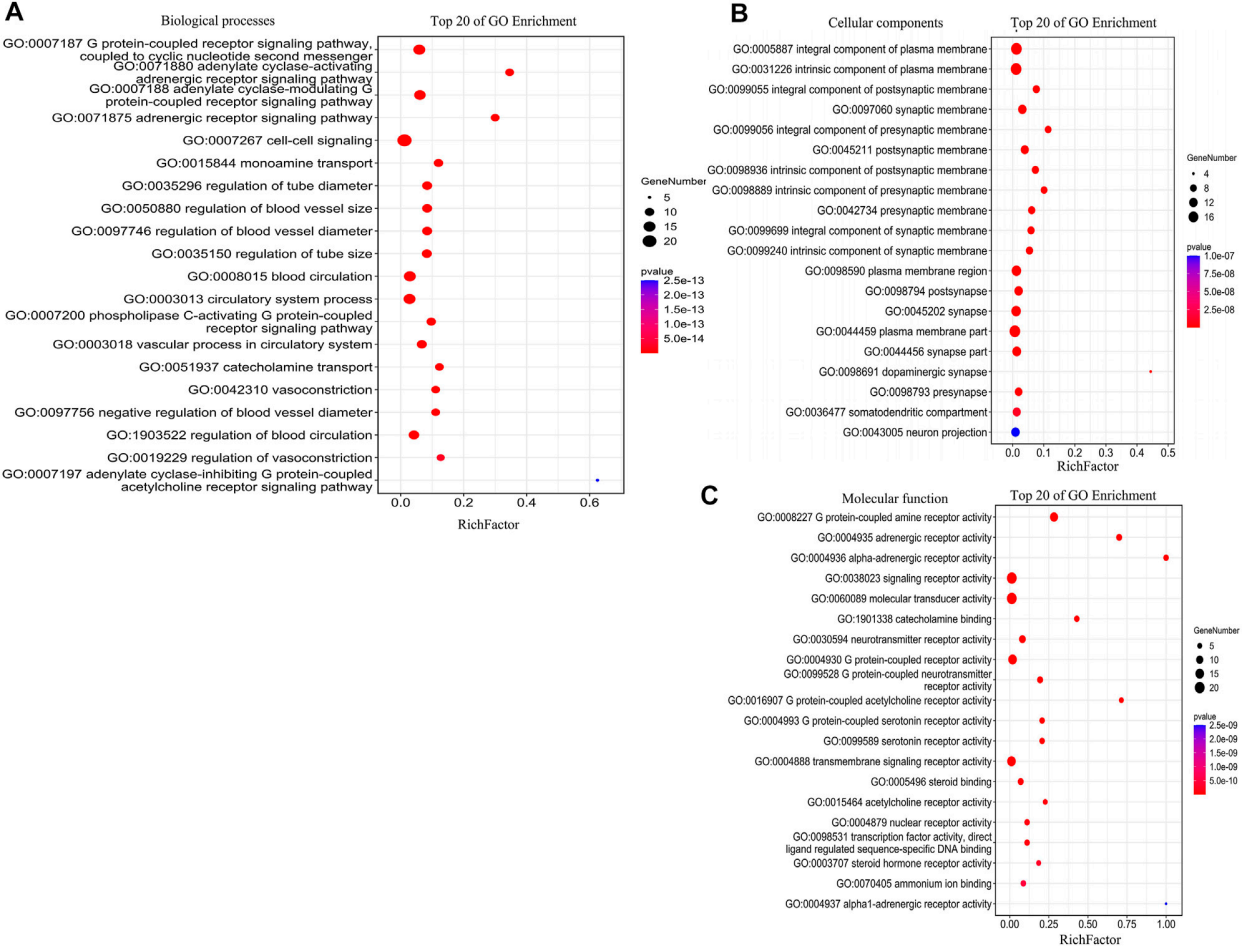
GO enrichment bubble plot analysis for TCM active ingredient–disease intersection targets (BP, CC, and MF). **(A)** Analysis of biological processes. **(B)** Analysis of cellular components. **(C)** Analysis of molecular function. The x-axis is the RichFactor; the y-axis is the GO Term. The point size indicates the number of gene, and the color of the points is the most important, representing the *p-*value. The degree of enrichment is proportional to the size of the circle and inversely proportional to the *p-*value (the closer to the red, the higher the degree of enrichment).

**FIGURE 7:**
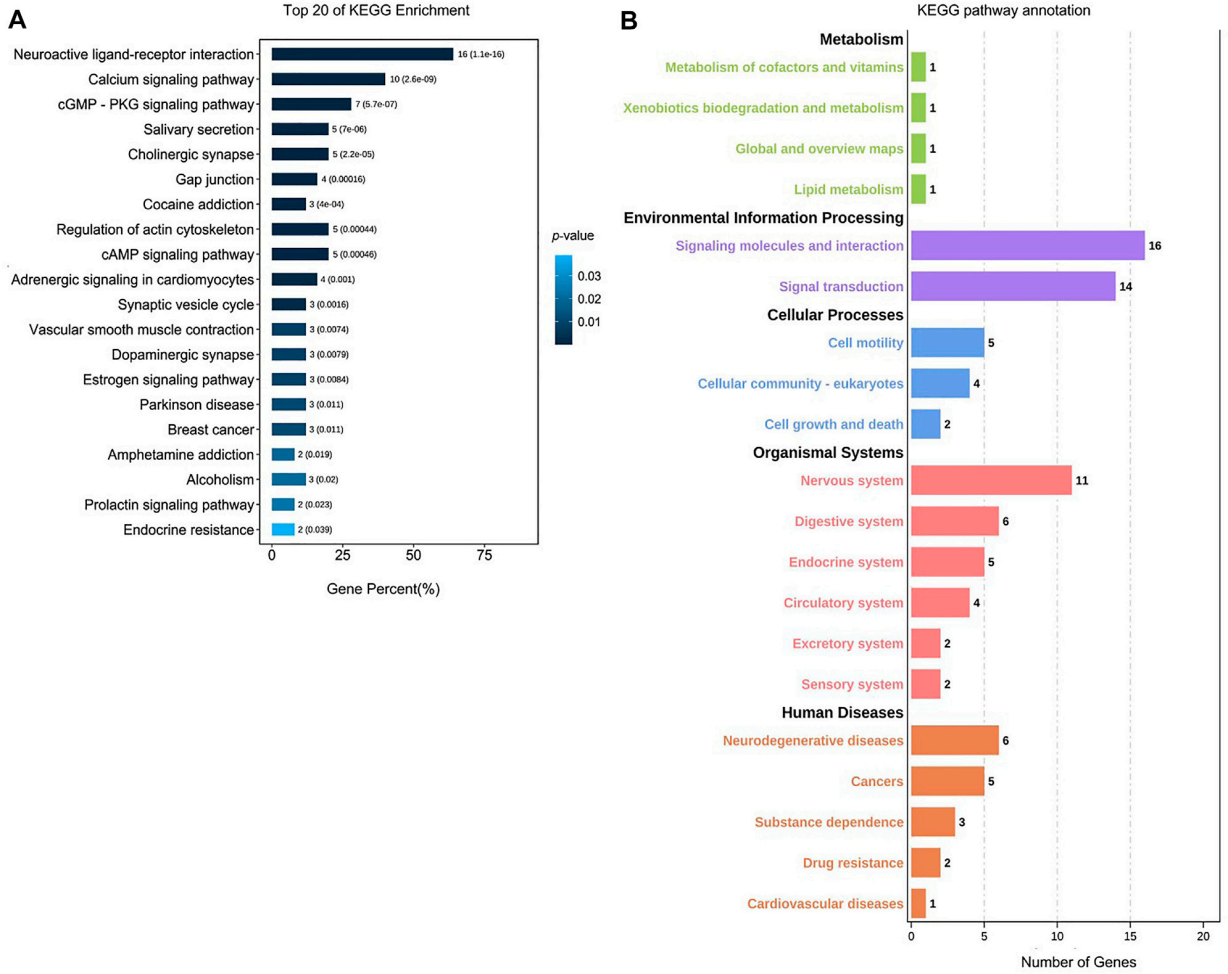
Analysis of the KEGG pathway for TCM active ingredient–disease intersection targets. **(A)** Analysis of the top 20 KEGG enrichment pathways. The x-axis is the gene percent (%), and the y-axis is the pathway’s name. The color represents value; the degree of enrichment is inversely proportional to the *p-*value (the closer the dark blue, the higher the degree of enrichment). **(B)** KEGG pathway annotation. The x-axis is the number of genes. The y-axis is the pathway name (green represents metabolic, purple represents environmental information processing, blue represents the cellular processes, red represents organismal systems, and orange represents human diseases).

To analyze the biological classification of herb ingredient–disease intersection targets, the GENE DENOVO platform was used to import the data for 26 intersection targets for GO enrichment and KEGG pathway analyses. Figures 6, 7 illustrate the specific results. As shown in Figure 6, the GO enrichment analysis identified twenty results with low *p-*values and increased target enrichment and divided them into three categories: biological process, cell composition, and molecular function. In BP analysis, the targets included the degree of target protein enrichment related to the G protein–coupled receptor signaling pathway, adenylate cyclase–activating adrenergic receptor signaling pathway, adrenergic receptor signaling pathway, monoamine transport, and other biological processes related to G protein coupling and cell signaling. In the CC type, the targets were mainly concentrated in the integral component of the plasma membrane, an intrinsic component of the plasma membrane, an integral component of the postsynaptic membrane, the synaptic membrane, and the target proteins involved in the cell composition of the plasma membrane and synapse. In the MF, the targets were mainly concentrated in G protein–coupled amine receptor activity, adrenergic receptor activity, alpha-adrenergic receptor activity, and molecular transducer activity, among which the molecular functions such as G protein–coupled amine receptor activity, signal transduction, and receptor activation were involved. The target protein is relatively abundant. The specific findings suggest that the herbs often used to treat PMS/PMDD may have a therapeutic effect by modulating these biological activities associated with the development of PMS/PMDD disorders.

### KEGG Pathway Analysis of the Active Ingredients of Herb–Disease Intersection Target

The KEGG pathway analysis results identified 52 pathways, as shown in Figure 7, including neuroactive ligand–receptor interaction (16%), calcium signaling pathway (10%), cGMP-PKG signaling pathway (7%), salivary secretion (5%)), cholinergic synapse (5%), gap junction (4%), cocaine addiction (3%), regulation of actin cytoskeleton (5%), cAMP signaling pathway (5%), adrenergic signaling in cardiomyocytes (4%), and other pathways. Among them, neuroactive ligand–receptor interaction (16%) and calcium signaling pathway (10%) had a higher proportion of genes than other pathways, and the *p-*value was much lower than that of other pathways. As a result, it can be concluded that herbs are more likely to treat PMS/PMDD. These pathways play a therapeutic role. According to the KEGG pathway annotation, the herbs predominantly exert their intervention impact on PMS/PMDD *via* signal molecular conduction, molecular interaction, the neurological system, and the endocrine system.

### Common Target Screening, GO Enrichment, and KEGG Pathway Analysis of 10 Herbs Used to Treat PMS/PMDD

The Venn diagram drawing tool was used to screen the ten herb co-action targets, including carboxylesterase 2 (CES2), kinase inserts domain receptor (KDR), sex hormone-binding globulin (SHBG), anaplastic lymphoma kinase (ALK), ESR1, ESR2, and acetylcholine. Supplementary Table S9 presents information on 27 frequently occurring targets, including esterase (ACHE) and butyrylcholine esterase (BCHE).

**FIGURE 8:**
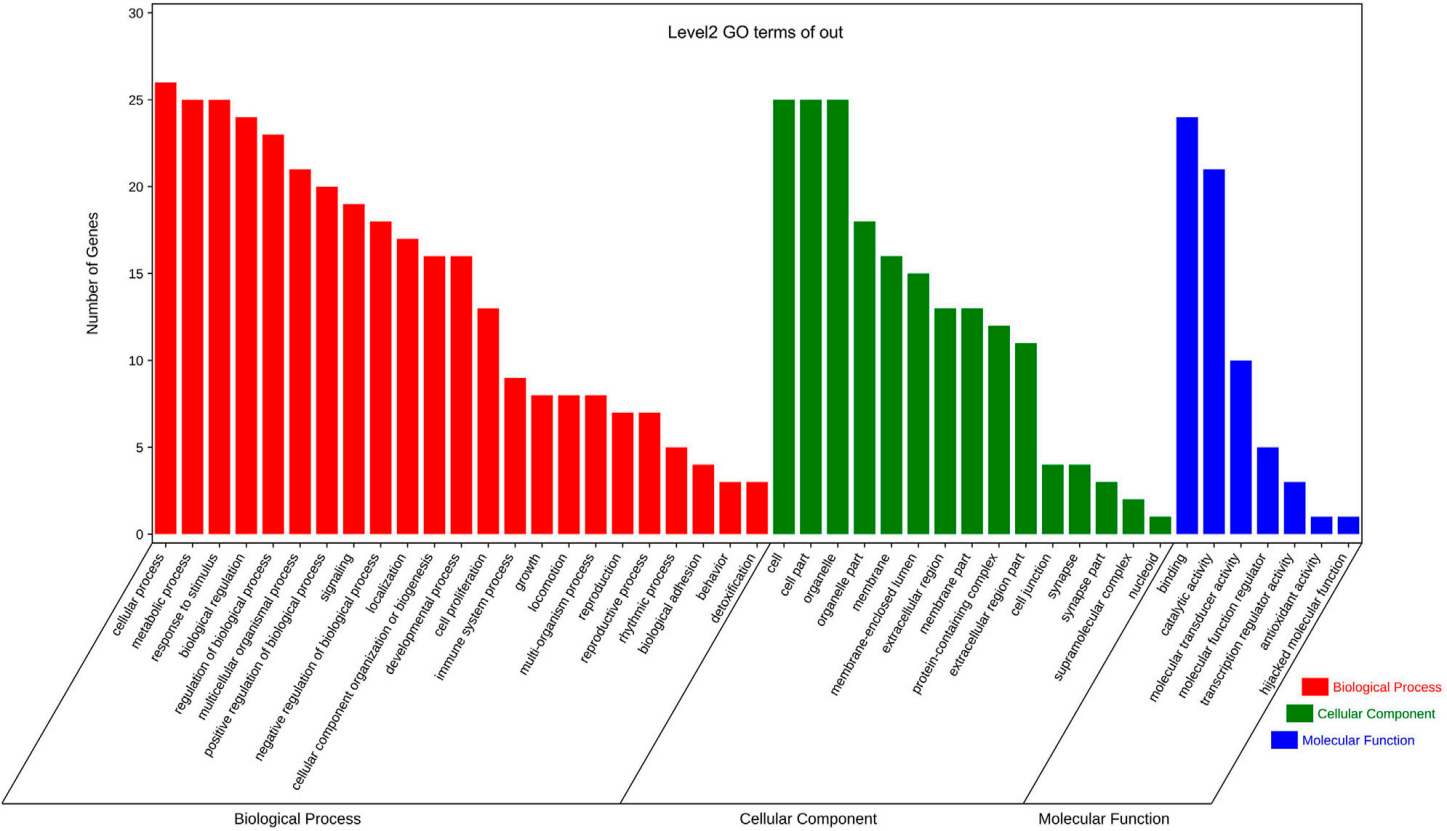
GO enrichment analysis of the predicted targets of 10 common herbs. The x-axis is the three functional groups (BP, CC, and MF) and the y-axis is the number of genes. Red represents the analysis of biological processes, among which the three functional groups, cellular process, biological regulation, and response to stimulus, contain the largest number of genes; green represents the analysis of cellular components, and cell and cell parts also account for more; blue represents molecular function enrichment analysis, binding, and molecular transducer activity functional group dominates absolutely.

**FIGURE 9:**
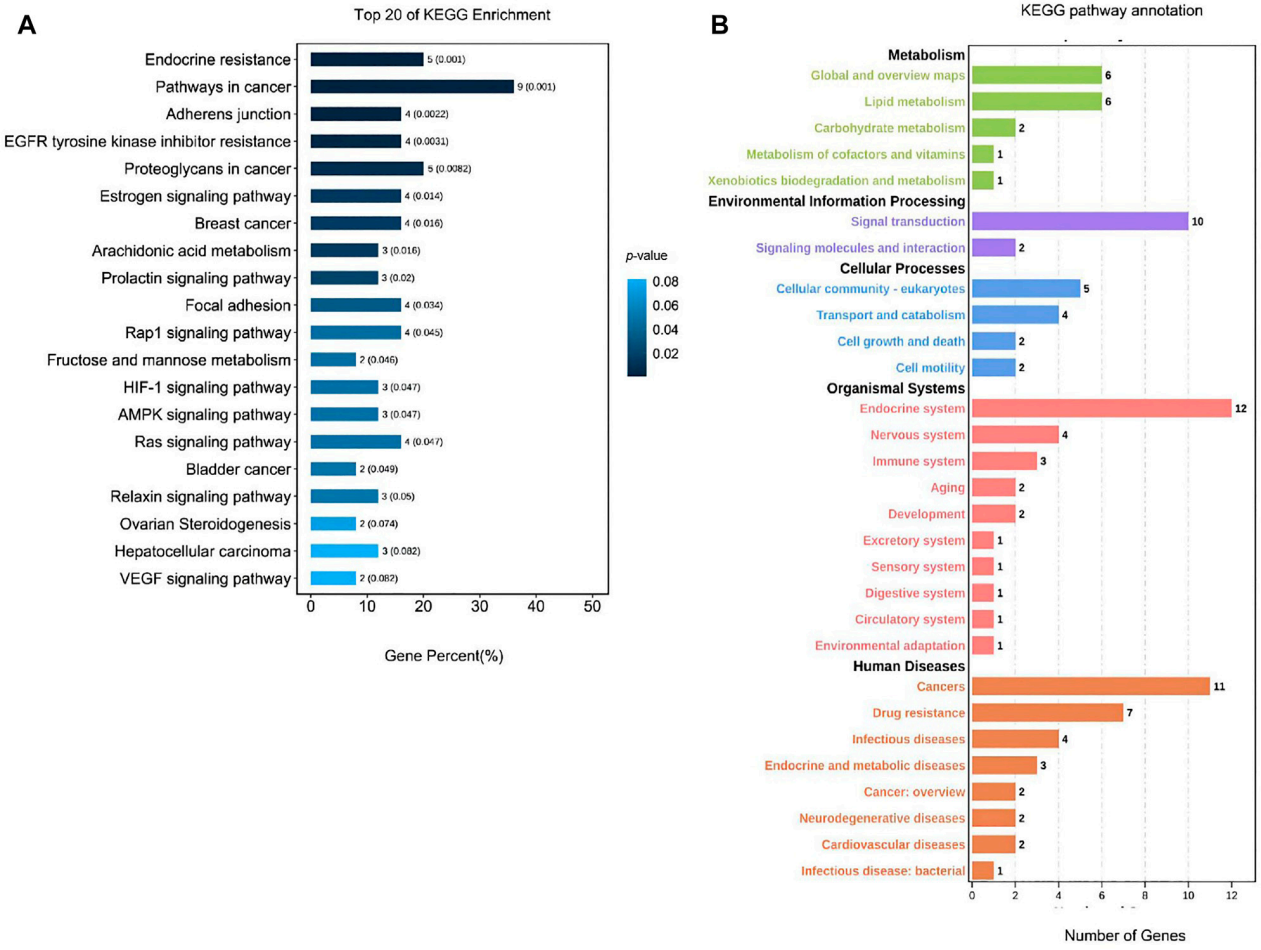
KEGG pathway analysis of the predicted targets of 10 common herbs. **(A)** Analysis of the top 20 KEGG enrichment. X-axis is the gene percent (%) and the y-axis is the pathway’s name. The color represents value; the degree of enrichment is inversely proportional to the *p-*value (the closer the dark blue, the higher the degree of enrichment). **(B)** KEGG pathway annotation. The x-axis is the number of genes. The y-axis is the pathway name (green represents metabolic, purple represents environmental information processing, blue represents the cellular processes, red represents organismal systems, and orange represents the human diseases.).

As seen in Figure 8, the GO enrichment analysis identified biological processes, cell components, and molecular functions with lower *p-*values and more significant target enrichment. In BP analysis, the cellular processes, the metabolic processes, response to stimulus, biological regulations, etc., were all involved. Among them, the highly enriched target proteins are involved in cellular metabolism and stress response. The target proteins involved in cell, organelle, membrane, protein-containing complex, synapse, and other cell compositions were enriched in the CC type. In the MFs, the target proteins involved in binding, catalytic activity, molecular transducer activity, molecular function regulator, transcription regulator activity, and other molecular functions were highly enriched. The KEGG pathway analysis results yielded 114 pathways, mainly related to endocrine resistance (5%), pathways in cancer (9%), adherens junction (4%), EGFR tyrosine kinase inhibitor resistance (4%), proteoglycans in cancer (5%), estrogen signaling pathway (4%), focal adhesion (4%), and other pathways, as shown in Figure 9. According to the KEGG pathway annotation analysis, the signaling pathways involved in the aforementioned Chinese medicines are primarily involved in lipid metabolism, cellular processes, signal transduction, neural and endocrine system regulation, and other connections and play a role in the occurrence of endocrine, metabolic, and neurodegenerative diseases.

## Discussion

In recent years, the incidence of PMS/PMDD has been on the rise. This condition has a significant impact on the body, psychology, and life of women of childbearing age. This condition has attracted much research attention worldwide. It is mainly treated using serotonin reuptake inhibitors (SSRIs) such as fluoxetine and sertraline. However, these drugs are not sufficiently active due to poor clinical differentiation of the disease. In addition, they are associated with side effects (Xue, 2017). Traditional Chinese medicine integrates with modern medicine based on the basic pathogenesis of liver failure and drainage. It divides PMS/PMDD clinical patients into liver qi inverse and liver qi stagnation subtypes. It has been reported that this approach achieves better curative effects in patients with liver diseases (Sun, 2016). This is because TCM can differentiate the syndrome and allow precise treatment. However, the mechanism of action for TCM should be investigated.

### Data Mining and Analysis of Commonly Used Chinese Medicine Prescriptions, Chinese Patent Medicines, and Single Medicines for the Treatment of PMS/PMDD for the First Time

For the first time, this study systematically summarized findings from previous studies on the treatment of PMS/PMDD using TCM. The TCM prescriptions were sorted according to standard clinical syndromes, treatment methods, and commonly used single medicines, and their pharmacological mechanisms were analyzed. This is the first scientific and systematic evaluation of prescription composition, medication rules, and treatment principles of PMS/PMDD in TCM treatment. Several types of PMS/PMDD syndrome differentiation have been reported, such as stagnation of liver qi, stagnation of liver qi to transform fire, and stagnation of qi and blood stasis. Stagnation of liver qi is the most commonly used method to diagnose and treat Xiaoyao San and Chaihu-Shugan-San (Zhao, 2011). Xiaoyao San was initially formulated in the Song Dynasty and was found to soothe the liver, relieve depression, nourish the blood, and improve spleen function. The Xiaoyao San prescription and other Chinese medicine are applied together, simultaneously regulating qi and blood in the liver and spleen. The experiments have proven that Xiaoyaosan can treat PMS/PMDD patients with liver qi stagnation without causing significant side effects. In addition, it has a low recurrent rate. Chaihu-Shugan-San comes from “The Complete Book of Jingyue.” The three flavors of *Rhizoma Chuanxiong*, *Rhizoma Cyperi*, and *Citrus aurantium L* are incorporated in Sini San to improve the spleen and qi. It is used to diagnose and treat chest tightness, irritability, and flank caused by liver qi stagnation. The herbs in the prescription can soothe the liver, regulate qi, and relieve depression (Zhu and Li, 2010). Xue XL (Xue, 2017) and others found that Chaihu-Shugan-San has long-range curative effects on PMS/PMDD patients with liver qi stagnation. Moreover, it did not show serious side effects. Hence, the stagnation of the liver will give serious consequences over time. *Cortex Moutan* and *Gardenia jasminoides Ellis* are added to clear away heat. Danzi Xiaoyao San is often used for treatment (Ju, 2009). For another common type - qi stagnation and blood stasis syndrome, Xuefu Zhuyu decoction is often used to treat this syndrome (Qiu, 2004.). Therefore, various prescriptions of TCM treat different subtypes of PMS/PMDD through syndrome differentiation and obtained good therapeutic effects. Other proprietary Chinese medicines, Jingqianshu Granules (An, 2016), Jingqianping Granules (Qiao et al., 2002), Shuyu Capsules (Li, 2016), and Xuefu Zhuyu Capsules (Bi, 2006), are also commonly used in the clinical treatment of different subtypes of PMS/PMDD. *Bupleurum Chinensis DC*, *Radix Paeoniae Rubra*, and *Angelica Sinensis* are the top three most common single herbs used to treat PMS/PMDD. According to the frequency ratio of herb attribution meridian, at present, the principal meridians of TCM in treating PMS/PMDD are three meridians of the liver, spleen, and lung, among which the liver meridian is the most frequently used PMS/PMDD treatment. This is consistent with the rule of TCM treatment of PMS/PMDD from the liver theory.

### Network Pharmacological Analysis of 10 Herbs Commonly Used in the Treatment of PMS/PMDD and Disease Intersection Targets

The construction of a typical intersection target network of herb ingredients and diseases can reveal new ideas for treating PMS/PMDD using TCM. In this study, we screened ten common targets from the intersection of single medicines and PMDD disease, and 26 targets were identified, among which ESR1 and ESR2 were the common targets of the ten single medicines and diseases. Many targets belonged to the solute carrier family 6, cholinergic receptors, adrenergic receptors, estrogen receptors, and dopamine receptor families, see Supplementary Table S6. The main pathways identified were neuroactive ligand–receptor interaction, calcium signaling pathway, cGMP-PKG signaling pathway, cAMP signaling pathway, and cholinergic synapse. Looking at the results of previous PCR chip research, we found that ADRA1A/ADRA2A/ADRB1/ADRB3 and SLC6/2/3/4 are abnormally expressed in the hippocampal brain region of the PMDD rat model (unpublished). These targets are all attributable to the adrenergic receptor family and the solute carrier family 6 (Slc6), consistent with ADRA1A/ADRA2A/ADRB1 and SLC6A1/11/12/13 of the same family screened out in this study. At the same time, the target involves multiple families, such as the estrogen receptor, and is also highly consistent with previous research results. Recent studies have found that some members of these families contribute to the pathogenesis of PMDD. For example, it was reported that genetic variation of ESR1 and ESR2 genes influenced susceptibility to PMDD (Huo et al., 2007). Other targets such as ADRA1A, ADRB1, and SLC6A4 modify the occurrence of depression and other mental diseases (Cheng et al., 2012; Rivero et al., 2014; Baudry et al., 2019).

On the other hand, we found several intersecting targets for PMS/PMDD intervention in the active ingredients stigmasterol, kaempferol, hyndarin, and other active ingredients. In Nasiara Karim’s study, stigmasterol may be a new steroid for treating neurological disorders through the regulation of GABA receptors (Karim et al., 2021). It was also found that stigmasterol can reduce the apoptosis of nerve cells through antioxidant stress and realize the protection of nerve cells (Pratiwi et al., 2021). Yan has shown *in vivo* that the kaempferide (KPF-4′-methyl ether) (C16H12O6), another glycosylated kaempferol derivative, improves the expression of the brain-derived neurotrophic factor (BDNF) responsible for the formation of the neural networks and neuronal plasticity (Yan et al., 2019). The existing studies on the mechanism of PMS/PMDD have found that the occurrence and development of PMS/PMDD are closely related to the GABAergic system and BDNF, which also provides a potential basis for stigmasterol and kaempferol for the treatment of PMS/PMDD (Sun et al., 2011; Song et al., 2016; Liu et al., 2016), We reasonably speculated that these ingredients might play an important regulatory role in the treatment of PMS/PMDD.

### Analysis of GO Enrichment

Comprehensively analyzing the results of GO enrichment, the intersection targets involved a wide range of molecular functions, diverse biological processes, and rich cell composition. The study found that G protein–coupled receptor, adrenergic receptor, and their corresponding signaling pathways are related to PMS/PMDD, which is consistent with the current research results on the mechanism of PMS/PMDD. A preliminary experimental study has proven that the occurrence and development of PMS/PMDD are closely related to the function of the ion channel coupled with GABAB receptor G protein and the conduction disorder of the signal pathway (Cai, 2009). Qiao detected neurotransmitter indicators in patients with PMS/PMDD and found that the level of norepinephrine (NE) in the urine of patients in PMS/PMDD was higher than that of the average population. The adrenaline level was lower than that of the average population (Qiao et al., 2007). These studies confirmed that herbs often used to treat PMS/PMDD may have a therapeutic effect by modulating these differential genes associated with the development of PMS/PMDD disorders.

### Analysis of KEGG Enrichment

According to the KEGG pathway analysis, we found that TCM can treat PMS/PMDD by regulating the neuroactive ligand–receptor interaction, calcium signaling pathway, and other signaling pathways. Previously, it was found that Baixiangdan capsules improved PMS/PMDD symptoms by regulating signal pathways such as neuroactive ligand–receptor interactions, mitogen-activated protein kinase, calcium, and gamma-aminobutyric acid signal transduction (Wei et al., 2018). Elsewhere, paeoniflorin (the main active ingredient of Paeonia lactiflora extract and Shuyu capsule) was found to regulate the Cav1.2-mediated the CaM/CaMKII signaling pathway. Paeoniflorin can significantly inhibit the intracellular Ca^2+^ concentration and Cav1.2 increase in current density induced by KCl. In addition, it showed beneficial effects against affective disorders by regulating Cav1.2 through the CaM/CaMKII pathway (Song and Xue, 2017). The abovementioned results further verified the correctness of the analysis results of the intersection targets of Chinese medicines and diseases in this study and clarified the Chinese medicine’s target and pharmacological mechanisms used in the treatment of PMS/PMDD. At the same time, this study also screened out some potential intervention targets and signal pathways through network pharmacological analysis, which can be used as the focus of the next step.

### Network Pharmacological Analysis Common Targets of 10 Therapeutic PMS/PMDD Herbs

We used the Venn diagram tool to analyze the ten herb targets for PMS/PMDD treatment. A total of 27 common targets such as PFKFB3, CES2, and KDR were obtained. Subsequently, the KEGG pathway analysis was performed on these targets, which identified 27 common targets (Supplementary Table S9). Among these targets, ESR1/ESR2 was the only target similar to the selected herb ingredient–disease intersection target (Supplementary Table S6). The current research on PMS/PMDD has not investigated the remaining targets. Of note, the genetic variation in KDR may be associated with the occurrence of recurrent depressive disorder (RDD) (Gałecki et al., 2013). Studies have found that VEGF induces antidepressant effects through the KDR-related signaling pathway. Thus, blocking KDR and receptor antagonists will reduce the effect and efficiency of VEGF (Nowacka and Obuchowicz, 2012). Therefore, we speculated that the 27 common targets may be potential or indirect targets for treating PMDD using TCM. Follow-up studies are needed to determine further the role of the abovementioned targets and the associated pathways in PMDD.

In summary, the pathogenesis of PMS/PMDD is complicated. TCM treatment can adjust the treatment focus through the compatibility of TCM or the changes in the taste and quantity of the herb to better treat different syndromes or concurrent syndromes of the same syndrome. TCM has shown to be flexible and accurate in application than Western medicine. In addition, TCM is safer, has fewer side effects, and has high clinical significance. However, it is worth noting that there is no unified approach to differentiate PMS/PMDD in TCM. Research on the antidepressant effects of TCM prescriptions and single herbs has not achieved much attention. Therefore, advanced methods based on systematic analysis should be adopted in future studies to improve the application of TCM in PMS/PMDD.

## Data Availability

The original contributions presented in the study are included in the article/Supplementary Material; further inquiries can be directed to the corresponding author.
